# Thiolated 2-Methyl-β-Cyclodextrin as a Mucoadhesive Excipient for Poorly Soluble Drugs: Synthesis and Characterization

**DOI:** 10.3390/polym14153170

**Published:** 2022-08-03

**Authors:** Brunella Grassiri, Andrea Cesari, Federica Balzano, Chiara Migone, Gergely Kali, Andreas Bernkop-Schnürch, Gloria Uccello-Barretta, Ylenia Zambito, Anna Maria Piras

**Affiliations:** 1 Department of Pharmacy, University of Pisa, Via Bonanno 33, 56126 Pisa, Italy; brunella.grassiri@phd.unipi.it (B.G.); chiara.migone@farm.unipi.it (C.M.); ylenia.zambito@unipi.it (Y.Z.); 2 Department of Chemical Sciences, University of Padova, Via Marzolo 1, 35131 Padova, Italy; andrea.cesari@unipd.it; 3 Department of Chemistry and Industrial Chemistry, University of Pisa, Via Moruzzi 13, 56124 Pisa, Italy; federica.balzano@unipi.it (F.B.); gloria.uccello.barretta@unipi.it (G.U.-B.); 4 Center for Chemistry and Biomedicine, Department of Pharmaceutical Technology, Institute of Pharmacy, University of Innsbruck, Innrain 80/82, A-6020 Innsbruck, Austria; gergely.kali@uibk.ac.at (G.K.); andreas.bernkop@uibk.ac.at (A.B.-S.); 5 Interdepartmental Research Centre “Nutraceuticals and Food for Health”, University of Pisa, 56100 Pisa, Italy

**Keywords:** cyclodextrin, microwave, nanoaggregates, mucoadhesion, dexamethasone, thiol, microrheology

## Abstract

Thiolated cyclodextrins are structurally simple mucoadhesive macromolecules, which are able to host drugs and increase their apparent water solubility, as well as interact with the mucus layer prolonging drug residence time on the site of absorption. The aim of this study was to synthesize through green microwave-assisted process a freely soluble thiolated 2-methyl-β-cyclodextrin (MβCD-SH). Its inclusion complex properties with dexamethasone (Dex), a poor water soluble drug, and mucoadhesive characteristics were also determined. The product was deeply characterized through NMR spectroscopy (2D COSY, 2D HSQC, 1D/2D TOCSY, and 1D ROESY), showing a thiolation degree of 67%, a selective thiolation on the C_6_ residues and a monomeric structure. The association constant of MβCD and MβCD-SH with Dex resulted in 2514.3 ± 32.3 M^−1^ and 2147.0 ± 69.3 M^−1^, respectively, indicating that both CDs were able to host the drug. Microrheological analysis of mucin in the presence of MBCD-SH showed an increase of complex viscosity, G′ and G″, due to disulphide bond formation. The cytotoxicity screening on fibroblast BALB/3T3 clone A31 cells indicated an IC_50_ of 27.7 mg/mL and 30.0 mg/mL, for MβCD and MβCD-SH, respectively. Finally, MβCD-SH was able to self-assemble in water into nanometric structures, both in the presence and absence of the complexed drug.

## 1. Introduction

New functional excipients and pharmaceutical techniques are joint in the developments of high performing drug delivery solutions [1,2,3]. Out of the several bioavailability limitations of topically administered drugs, poor solubility and short retention time on the absorption membrane play crucial roles. 

As widely evidenced in the literature, targeting mucus could be used as a valuable tool for increasing the drug residence time on mucosal surfaces [4]. Briefly, mucus is mainly made of water, salts, and various glycoproteins, primarily mucins, responsible for protecting the epithelium from mechanical and enzymatic damage. Mucus covers various epithelia including the pulmonary, gastrointestinal and ocular mucosa. Each mucosal barrier has its peculiarity; mucosal surfaces are targets for local therapies but are also useful for achieving a non-invasive administration of systemic drugs [5]. Mucins are the most abundant glycoproteins in the mucus layer rich of moieties that are ionized at physiological pH, and therefore can interact through an H-bond, ionic interactions, chain entanglements or hydrophobic interactions with functionalized excipients. Moreover, mucins are notably rich in cysteines substructures. In recent years, attention has been paid on macromolecular excipients, mainly of natural origin as they are biodegradable and biocompatible, in addition to having good viscosity and mucoadhesive characteristics [6,7]. Thiomers represent an innovative class of mucoadhesive polymers, capable of forming disulphide bonds by thiol/disulphide exchange reactions with these cysteine residues of mucins, ensuring stronger mucoadhesion because of covalent bonds [8,9]. 

An efficient mucoadhesive drug delivery system must, on the one hand, adhere on the mucus gel layer and on the other hand facilitate the release/permeation of the drug from the formulation [4]. Therefore, growing interest has been reported in mucoadhesive thiolated cyclodextrins, as they are valuable solubilizing agents as well as mucoadhesive tools for topical drug delivery of poorly soluble or easily degradable drugs [10]. Adhering to the mucus layer through disulphide bond formation thiolated cyclodextrins efficiently improves drug residence time and permeation when topically administered, as demonstrated in vitro [11,12,13] and in vivo [14]. Previously described thiolation methods are based on organic solvents. Moreover, most of the synthesis require several steps of reaction and workup, being hardly scalable [10].

In addition to the encouraging results obtained on the ocular application of thiolated hydroxypropyl-β-cyclodextrin (HPβCD-SH) [14], the aim of the present study is to evaluate the potential of thiolated 2-methyl-β-cyclodextrin (MβCD-SH) as a functional mucoadhesive drug complexing agent. Indeed, methyl-β-cyclodextrin is a freely soluble βCD derivative, with pharmaceutical interest and approved in products with ocular and nasal routes of administration, being at present the only cyclodextrin derivative used as an excipient for nasal delivery (EMA 2017). A CD selectively methylated in the C_2_ position was used to optimize the microwave assisted synthesis process, in the complete absence of organic solvents. Indeed, microwave assisted synthesis was demonstrated to be more efficient, more selective and provides, in most cases, a higher yield than conventional reactions at high temperatures [15]. The product was deeply characterized through NMR investigation as well as in terms of mucoadhesion, cytotoxicity, complexing capability toward dexamethasone (Dex), and as for the formation of self-assembled nanoaggregates in aqueous solutions. 

## 2. Materials and Methods

### 2.1. Materials 

Deuterated water (D_2_O, 99.9%) and dimethyl sulfoxide (DMSO-d_6_, 99.9%) were purchased from Deutero GmbH (Kastellaun, Germany). Additionally, 2-Methyl- β-cyclodextrin (MβCD) was kindly provided by Roquette Italia (Alessandria, Italy; MW 1191 g/mol, degree of C_2_ substitution ~0.5, corresponding to ~4 methyl groups per cyclodextrin molecule). Dexamethasone (Dex), acetic acid, thiourea (≥99.0%), hydrochloric acid 37%, acetone (≥99.0%), Sephadex^®^ G-15 resin and mucin from porcine stomach type II were purchased from Merck (Darmstadt, Germany).

The fibroblast BALB/3T3 clone A31cell line was obtained from American Type Culture Collection, Dulbecco’s Modified Eagle’s Medium (DMEM), supplemented with 2 mM L-glutamine and 1% penicillin/streptomycin and 10% calf bovine serum, trypsin and ethylenediaminetetraacetic acid (EDTA) were obtained from Merck (Darmstadt, Germany).

### 2.2. Instruments

NMR measurements were performed on a Varian INOVA600 spectrometer (Varian Inc., Palo Alto, CA, USA) operating at 600 MHz for ^1^H. The spectra are referenced through the solvent lock (^2^H) signal according to the IUPAC recommended secondary referencing method. The temperature was controlled to 25 ± 0.1 °C through a Varian control unit. In addition, 2D NMR spectra were obtained by using standard sequences. Spectral width used was the minimum required in both dimensions. Then, 2D gCOSY (gradient Correlated Spectroscopy) spectra were recorded in the absolute mode acquiring 16 scans with a 1 s relaxation delay between acquisitions and 4k data points for each of 200 FIDs, followed by 2D TOCSY (Total Correlation Spectroscopy) spectra, which were recorded acquiring 8 scans with a 1 s relaxation delay, 200 increments, 4k data points and a mixing time of 80 ms. Additionally, 2D gHSQC (gradient Heteronuclear Single Quantum Coherence) spectra were obtained with 1.2 s relaxation delay and 64 scans for each of the 200 increments, and 1D TOCSY spectra were recorded with a mixing time of 80 ms, a delay of 1 s and 512 scans. Then, 1D ROESY spectra were recorded with a mixing time of 300 ms, a delay of 1 s and 512 scans. DOSY (Diffusion Ordered Spectroscopy) experiments were carried out by using a stimulated echo sequence with self-compensating gradient schemes and 64k data points. In particular, gradient strength was varied in 15 steps (16 transients each) and delays Δ and δ were optimized in order to obtain an approximately 90% decrease in the resonance intensity at the largest gradient amplitude. After data acquisition, each FID was apodized with 1.0 Hz line broadening and Fourier transformed. The baselines of all arrayed spectra were corrected prior to processing the data. Gradient amplitudes in DOSY experiments have been calibrated by using a standard sample of D_2_O 99% (19 × 10^−10^ m^2^s^−1^).

### 2.3. Synthesis

Thiolated methyl-β-cyclodextrin (MβCD-SH) was prepared by optimizing the method reported in Grassiri et al., 2022 [14] (reaction conditions are reported in Table S1). Briefly, 200 mg of MβCD was dissolved in 1 mL of 10% acetic acid, whereas 1.07 g of thiourea were dissolved at 40 °C under stirring in 4 mL of HCl 0.44 M. Once both compounds were solubilized, the dissolved thiourea was added dropwise to the MβCD solutions. The resulting mixture was irradiated in a microwave device (Microonde Biotage Initiator) with a temperature-controlled setting and maximum power set at 90 Watt. The irradiation was carried out for 5 min at 87 °C and for one hour and 55 min at 80 °C. After this first step, a hydrolysis reaction was started by adding 10 M NaOH to reach a slightly basic pH (pH = 8–9). The resulting mixture was irradiated by MW for 3 min at 80 °C. The product was dried and subjected to 5 washing cycles with 20 mL of acetone, each followed by 20 min centrifugation (5.000 rpm; room temperature, Haraeus Megafuge 16 R—(Thermo Scientific, Waltham, MA, USA) and then lyophilized (VirTis lyophilizer, freezing temperature −40 °C, drying at 30–40 mTorr, up to 16 °C). Purification consisted of two column passages through Sephadex G-15 resin, size exclusion chromatography, with milli-Q water as the mobile phase. The resulting MβCD-SH product was lyophilized and stored at −20 °C. 

### 2.4. Determination of Thiol Content in MβCD-SH

The thiol content was determined by ^1^H NMR analysis in D_2_O. The areas assigned to the anomeric protons of thiolate and non-thiolated sugar residues were integrated and compared. The percentage of each integrated area on the sum of all the anomeric peaks areas was expressed as a % of thiolated sugars.

### 2.5. Determination of the Dex/Cyclodextrins Association Constant

The complex association constant (K_a_) was determined by spectrometric titration based on the Benesi–Hildebrand method [16] and performed by UV-VIS spectrometry. Two water stock solutions were prepared containing respectively: (a) Dex at its intrinsic maximum solubility in water 5.8 ± 0.13 µM, prepared starting from the 1 mg/mL suspension in water, let under left under stirring for 15 min and then centrifuged and filtered; (b) cyclodextrin 7.14 mM in water. At least 3 series of dilutions were prepared by mixing the Dex solution (a) with that of CD (b), so that the concentration of the guest substance (Dex) in the solution remained constant (0.97 µM) while increasing the CD content (range 0–5.95 mM). Samples were equilibrated for 72 h in a thermostatic shaking bath at 20 °C. UV-VIS spectra were acquired in the 200–450 nm range and the second derivative was calculated. In order to knock down the thiol interference, Dex/cyclodextrin interaction was evaluated at 290 nm (normalized to 450 nm).

### 2.6. Determination of Nanometric Aggregates

Milli-Q water solutions of MβCD and MβCD-SH, either plain or complexed with dexamethasone were analysed via dynamic light scattering (DLS; Zeta Sizer Nano series instrument) at 25 °C. Cyclodextrins were in the concentration range of 3–125 mg/mL. 

### 2.7. Microrheological Tests

The measurements were performed by using the Nano Zetasizer ZS instrument as previously reported [17,18]. The microrheological characterization of MβCD and MβCD-SH was performed using type II porcine gastric mucin. Type II porcine gastric mucin (3 mg/mL) was prepared in Milli-Q water and 0.45 µm filtered (cellulose acetate filters). A solution of 10 mg/mL of either MβCD or MβCD-SH was then prepared in deionized water and the microrheological evaluation was performed on samples having the following composition: 5 µL/mL of tracer (latex polystyrene particles, diameter 500 nm, Beckman), 7.38 mg/mL of MβCD or MβCD-SH, 0.3 mg/mL of mucin and 8.1 mg/mL of 5% NaCl. As a reference, 1 mL of sample was prepared, having the same composition but without MβCD or MβCD-SH. 

### 2.8. Cell Viability Assay

Fibroblasts were grown in a CO_2_ incubator (Heracell 150i series) at 37 °C and 5% CO_2_ with cells sub-cultured at 80–90% confluency. A sub-confluent monolayer of fibroblast BALB/3T3 clone A31 cells was trypsinized, centrifuged at 1000 rpm for 5 min and re-dispersed in the growth medium. Cells were seeded in 96-well plates at a seeding density of 8 × 10^4^ cells/well, and after 24 h, the medium was removed from each well and replaced with DMEM containing MβCD or MβCD-SH in a concentration range from 0.1% to 5% (*w*/*v*). After 4 h of incubation, media were removed and substituted with a fresh medium containing 10% WST-1 reagent solution and maintained for 4 h at 37 °C, 5% CO_2_. The formazan dye absorbance was quantified at 450 nm with the reference wavelength at 655 nm (BioTek 800/TS, Thermo Scientific, Waltham, MA, USA).

### 2.9. Statistical Data Analyses

Each analytical test was conducted at least in triplicate on each batch sample. When possible, the data sets were statistically compared by applying a Student *t*-test, and *p* < 0.05 and *p* < 0.01 were considered indicative of a significant difference.

## 3. Results and Discussion

### 3.1. Synthesis 

The synthesis was conducted in the complete absence of organic solvents. Several microwave (MW) conditions were tested (Table S1). The selection of optimal reaction conditions was set in terms of mass yield (%) and product thiolation degree (TD, % of thiolated sugars). In general, the product loss and the resulting low yields values are due to cyclodextrin degradation through ring-opening and fragmentation [14]. Initially, the reaction was performed as a single MW reaction step, but notably, a quick 5 min boost to a higher temperature (87 °C) was effective in improving the thiolation degree, without significantly altering the reaction yield. The extension of the II step to longer reaction times determined a greater improvement of TD, without additional product loss. Differently, the use of higher amounts of thiourea leads to a drastic drop in reaction yield, due to massive product degradation. In agreement with observations for the HPβCD, the application of milder conditions, in the presence of citrate buffer, was unsuccessful and nearly no thiolation was obtained. However, with respect to HPβCD [14], the conditions selected for the thiolation of MβCD led to a higher thiolation degree, 67% vs. 33% (for MβCD-SH and HPβCD-SH, respectively), and comparable product yields of about 30%.

### 3.2. NMR Characterization

**Figure 1:**
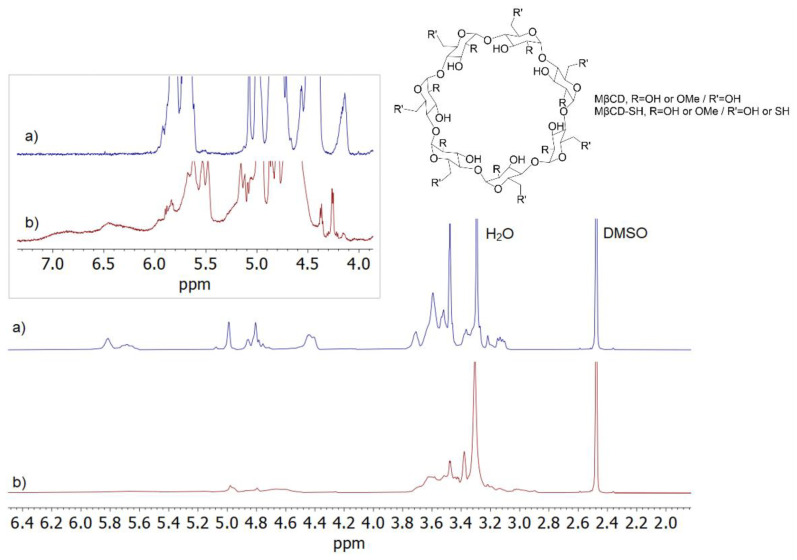
^1^H NMR (600 MHz, DMSO-d_6_, 25 °C, 5 mg/mL) spectra of: (**a**) MβCD, and (**b**) MβCD-SH.

Remarkably different spectral profiles of MβCD-SH and its precursor (MβCD) in DMSO-d_6_ (Figure 1), with a large distribution of signals for MβCD-SH, confirmed the occurrence of derivatization, producing a wide distribution of products. The NMR spectrum of MβCD-SH showed signals between 2.8–3.9 ppm due to ring protons and methoxy groups and several structured signals between 4.0–5.3 ppm, which were attributed to the resonances of the anomeric protons and primary hydroxyls. The signals between 5.4 ppm and 6.1 ppm were assigned to the secondary hydroxyls, whereas the resonances of -SH derivatizing groups on the primary sites produced very broad signals at frequencies higher than 6.1 ppm [11], which were not present in the NMR spectrum of MβCD. Moreover, the decrease of the integrated area of the spectral region including the superimposition of anomeric protons and primary hydroxyl groups (with respect to MβCD) roughly corresponded to the increase of the integrated area in the spectral region at 6.1 ÷ 7.2 ppm, in favour of the expected derivatization at the primary sites of the cyclodextrin (Figure S1).

**Figure 2:**
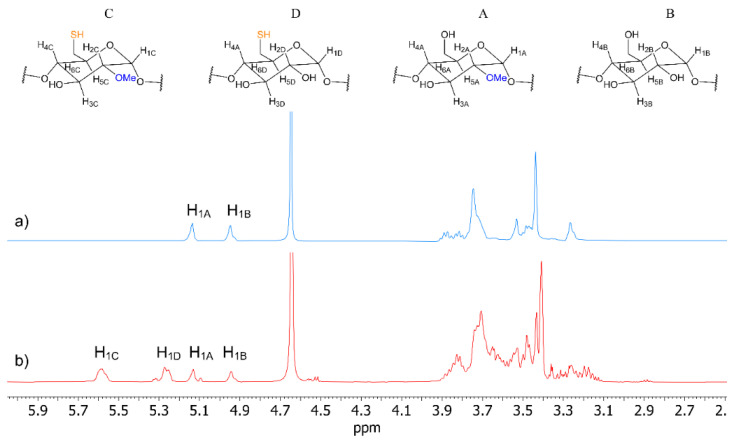
^1^H NMR (600 MHz, D_2_O, 25 °C, 5 mg/mL) spectra of (**a**) MβCD and (**b**) MβCD-SH.

In order to simplify the spectral profile of MβCD-SH, its characterization was performed in D_2_O, where -OH groups were not observed as the consequence of the -OH/-OD exchange (Figure 2). In water, four main clusters of signals were detected and attributed to four different kinds of glucopyranose units with different derivatizing groups (Figure 2). In particular, four resonances at 5.58 ppm, 5.26 ppm, 5.13 ppm, and 4.94 ppm were found, which correlated in the 2D HSQC map (Figure S2) with carbons at 96.1 ppm, 99.6 ppm, 99.1 ppm, and 101.6 ppm, respectively, to be unequivocally attributed to the four anomeric protons of the different units. Among the four anomeric protons resonances, the two more shielded ones at 5.13 ppm and 4.94 ppm were almost superimposable to those of MβCD precursor (Figure 2, Table 1), and hence were attributed to A and B (non-thiolated) units, methylated and non-methylated, respectively (for a more detailed characterization of MβCD, see ref. [19]). Therefore, the other two signals at 5.58 ppm and 5.26 ppm were attributed to C and D units, both bearing thiol derivatizing groups. As a matter of fact, only two main methoxy groups resonances (^13^C methyl resonance at 59.1 ppm and 58.4 ppm, HSQC in Figure S2) were detected at 3.43 ppm and 3.40 ppm, the first of which corresponded to the non-thiolated/methylated unit A. Therefore, the other one at 3.40 ppm was attributed to the thiolated/methylated unit C. Accordingly, in the 1D ROESY spectra (Figure S3), through-space dipolar correlations were detected between the methoxy resonance at 3.40 ppm and the anomeric proton at 5.58 ppm (H_1C_), whereas the other methoxy resonance produced dipolar interaction at the frequency of the anomeric proton at 5.13 ppm (H_1A_). The remaining ring protons of the four units were attributed starting from the assigned anomeric protons, on the basis of comparative analysis of homoscalar (COSY, 1D TOCSY), heteroscalar (HSQC) and dipolar homonuclear (1D ROESY) correlations (Table 1, Figures S2–S5).

By comparing the integrated areas of the anomeric protons of C + D units and A + B units (Figure S6), the thiolation degree of 67.4% was calculated, which reasonably arose from a large distribution of products as witnessed by the structured nature of the cluster signals. It is noteworthy that the ratio of the integrated areas D/B was larger than C/A, witnessing that non-methylated units are derivatized in a larger amount with respect to methylated ones (Figure S6).

The diffusion coefficient of MβCD-SH (D = 2.48 × 10^−10^ m^2^s^−1^, 5 mg/mL, D_2_O) was comparable to that one of MβCD (D = 2.61 × 10^−10^ m^2^s^−1^, 5 mg/mL, D_2_O), concluding that derivatized cyclodextrin is present as monomeric macrocycles with an irrelevant contribution from dimerization or polymerization processes due to -SH oxidation.

### 3.3. Complexation to Dex

**Figure 3:**
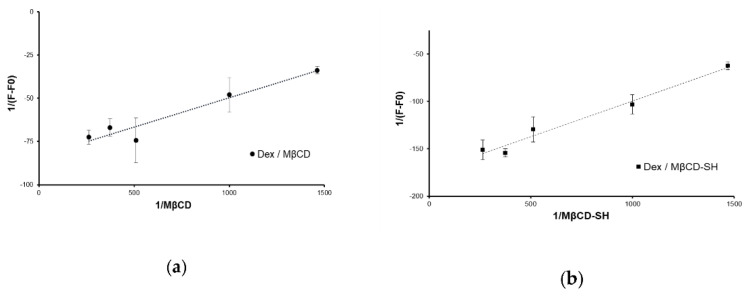
Benesi–Hildebrand plot for Dex/MβCD (**a**) and Dex/MβCD-SH (**b**). Each point is the mean ± standard deviation of 3 values.

Cyclodextrins are known to form inclusion complexes with lipophilic guest molecules, thus increasing the apparent solubility of the latter. A dynamic equilibrium between complexed and free forms, both water-soluble, is then established. The association constant of the Dex/MβCD complex (K_a_) was determined by the Benesi–Hildebrand method [16] in association with UV-VIS spectrometry by exploiting the variation in the absorption of solutions having the same Dex content and increasing concentrations of MβCD. The Ka was calculated through the Benesi–Hildebrand equation [20]:

1/(A − A_0_) = 1/(A′ − A_0_) × 1/[CD] + 1/(A′ − A_0_)

in which [CD] represents the concentration of cyclodextrin in solution, A and A_0_ are the absorbances, respectively, in the presence and absence of CD and A′ is the absorbance when all the Dex molecules are complexed with CD. The good linearity of the plot in Figure 3, highlighted by the high value of R^2^ equal to 0.923 and 0.979, allowed for the calculation of the complex constants. Both cyclodextrins were able to include dexamethasone, showing Ka values of 2514 ± 32 M^−1^ and 2147 ± 69 M^−1^ for Dex/MβCD and Dex/MβCD-SH, respectively. The obtained values are in line with the reported literature data [21,22] and suggest that the structural modification of the toroid does not affect the ability of cyclodextrin to form inclusion complexes.

### 3.4. Microrheological Evaluations

The mucoadhesive properties of MβCD-SH were evaluated by micro-rheology analysis (Table 2). It is firstly noted that MβCD presents lower values with respect to mucin control suspension, suggesting that MβCD could partially exert a fluidifying role towards mucins. In general, cyclodextrins do not significantly change the viscosity of mucus control samples [23]; however, cyclodextrin can act as a rheology modifier with respect to the interacting molecule [24]. The effect of thiol moieties is greatly reflected on the increasing values of both complex viscosity (η*) and G″ module, indicating a direct interaction between mucin and thiolated cyclodextrin molecules [12,17].

**Figure 4:**
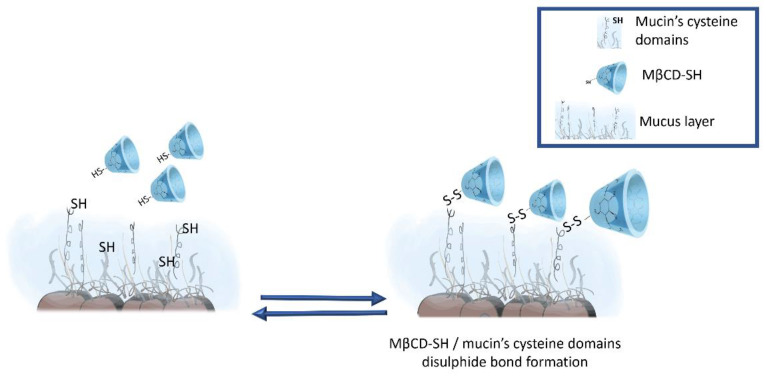
Mucoadhesion: representation of the molecular interaction mechanism of MβCD-SH derivative and mucins.

The interconnections seem to occur with mucus glycoproteins exhibiting cysteine-rich subdomains (Figure 4) typically involved in intra- and intermolecular disulphide bridge formations [25]. 

The maintenance of the mucoadhesive capability of the thiolated cyclodextrin is going to be preserved in the Dex/CD inclusion complex, since the modification is at the C6 moieties, pending at the smaller rim of the toroid and thus not perturbed by the complexing with the drug. The insertion of corticosteroids in β-CD has been already described and it is known that once the inclusion occurs, hydrophilic hydrogen bond interactions are present at the wide rim of the cyclodextrin, driving the deep inclusion of the drug into the apolar cavity [19]. As such, the smaller rim with pendant thiols is available for mucosal interaction, similarly to what occurs for free cyclodextrin [4].

### 3.5. Cell Viability Evaluations

**Figure 5:**
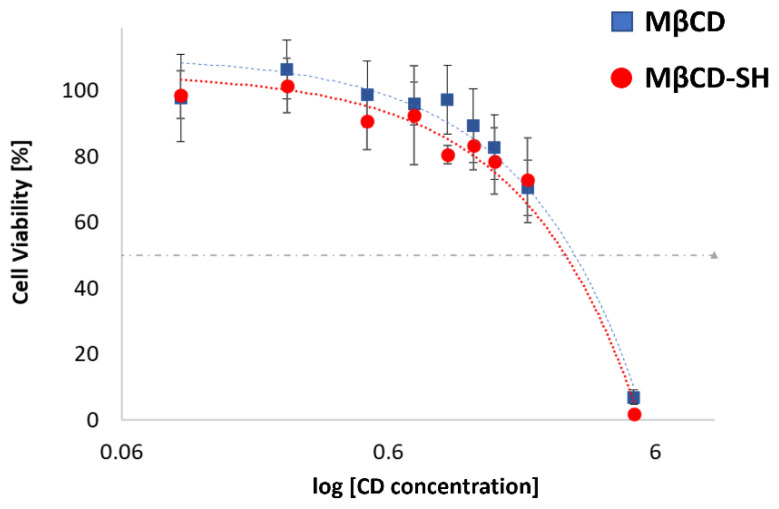
Cell viability screening performed on BALB/3T3 cell line clone A31, exposed for 24 h to MβCD or MβCD-SH in the 0.1–5% *w*/*v* concentration range. Untreated cells were used as control. The values indicated in the figure are means ± standard deviation of 6 replicates.

To assess the toxicity of native MβCD and MβCD-SH, a cytotoxicity screening was performed on the cell line BALB/3T3 clone A31, according to ISO 10993-5 [ISO 10993] for the biological evaluation of medical devices (Figure 5). The IC_50_ of the MβCD and its thiolated derivative were 27.7 mg/mL and 30.0 mg/mL, respectively. Therefore, the cytotoxicity of the synthetized product is not significantly affected by the functionalization.

It has been already verified that when the modified cyclodextrin preserves the cyto-compatibility of the native compound, the cyto-compatibility is also preserved with the inclusion complex [7]. Generally, the main concern is the toxicity of the drug more than that of the macromolecular carrier since the applied cyclodextrin is generally recognized as safe (GRAS). The structure of the cyclodextrin is not significantly altered by the inclusion complex, generally only slightly perturbed since the inclusion can make the toroid more rigid and increase its hydrophobicity. Since the complexation with dexamethasone favours the solubility of the drug linearly with the concentration of the cyclodextrin, there is not a significant alteration of the toroid, and no increased cytotoxicity is expected for the dexamethasone inclusion complex.

### 3.6. Determination of Nanometric Aggregates

The capacity of cyclodextrins to form nanometric aggregates has been the object of several studies [26,27,28]. Most of these studies though evaluate the capability of grafted and functionalized cyclodextrins to form self-assembly inclusion complexes. Natural αCD, βCD and γCD and derivative cyclodextrins such as HPβCD or HPγCD, both plain and complexed with hydrocortisone, have previously been shown to form spontaneous nanoaggregates in an aqueous solution at or above 1% to 10% *w*/*v* [26,29]. The dimension of such aggregates is between 10 and 100 nm [29]. Within this study, we evaluated the capability of cyclodextrins to self-aggregate in aqueous solutions in a concentration range between 0.3–12.5% *w*/*v*. Only MβCD-SH was able to form small nanoaggregates at all tested concentrations, whereas no aggregates were detected at any tested concentration of MβCD. MβCD-SH aggregates show a trend of dimensional increase at increasing concentrations, but the reported differences are not statistically significant. The size of the aggregates is reported in Table 3. The aggregates were detected only with DLS and not with NMR investigations. These results, apparently in discordance, are consistent with what already reported in the literature, regarding the detection of CD aggregate formation. CDs form transient clusters, which can rarely be observed within the detection limit of the NMR experiments [30]. Differently, DLS has been reported as a more sensitive method to detect self-association of CDs [31].

**Figure 6:**
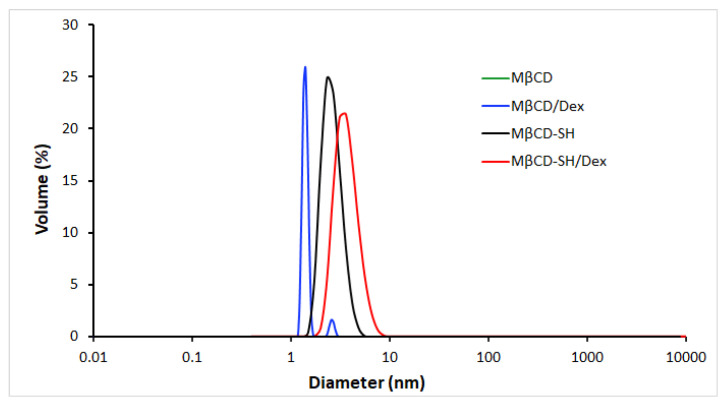
Particle size distribution in water of 0.3% cyclodextrin either plain (MβCD; MβCD-SH) and as inclusion complexes of Dex (MβCD/Dex; MβCD-SH/Dex). MβCD (green line) did not show any aggregate (no peak in the graph); MβCD/Dex (blue line) showed aggregates of 1.5 ± 0.13 nm; MβCD-SH (black line) showed aggregates of 2.5 ± 0.4 nm; MβCD-SH/Dex (red line) showed aggregates of 4.7 ± 1.2 nm.

Then, the dimension of the nanometric aggregates at the lowest concentration (0.3% *w*/*v*) was measured with the complexing drug and compared with the previously obtained results at 0.3% *w*/*v* (Figure 6).

MβCD is able to form nanoaggregates only in presence of Dex, and as described in the literature, the self-association process of CDs is a drug-induced and a concentration dependent effect [28]. Differently, MβCD-SH formed small nanoaggregates both as plain cyclodextrin and as inclusion complex with Dex. In aqueous solution, CDs are surrounded by a hydration shell deriving from the hydrogen bonds with the water molecules of the medium. However, when CDs concentration increases, the water molecules are replaced by other molecules of CDs to form transient clusters and water-soluble aggregates [31]. As seen from the NMR characterization, in the MβCD-SH molecule, the -OH in C¬_6_ position is replaced with -SH moiety, thus having a different behaviour in forming H-bridges. Sulphur is both a potential H-bond acceptor (either in its protonated or deprotonated states) and a very good H-bond donor. The strength of the H-bond depends on the hydrogen-bonding partner and its local environment, as demonstrated for thiol bearing compounds such as cysteine [32], and O−H···O hydrogen bonding is stronger than O−H···S hydrogen bonding, in some cases [33]. The lowest concentration at which CDs aggregate (similar to a critical aggregation concentration) is correlated to their ability to form hydrogen bonds with water [31]. For this reason, the different tendency of thiol moieties to form and sustain H-bonds could be a key element for the formation of MβCD-SH aggregates at lower concentrations than those for MβCD. The observed behaviour appears promising for the development of stable nanosized carriers based on the self-association of thiolated cyclodextrins.

## 4. Conclusions

Within this study, for the first time, thiolation and characterization of MβCD was carried out. The microwave-assisted synthesis method was quick and easy, carried out in the absence of organic solvents and suitable for a green scale up. The thiolation of the product was of 67%, verified via NMR characterization. This high thiolation degree on 2-methyl-β-cyclodextrin has to be considered of great impact as the CD is already functionalized in a reactive position. The thiolation was obtained selectively on C_6_, as it is the most reactive -OH toward such modification. The diffusion coefficient suggested that the product is a monomer; therefore, no oxidised disulphide bridges between different CDs were detected. The product maintained its high solubility in water, and efficiently formed complexes with the poorly soluble drug Dex. Interactions between cysteine domains of mucins and thiol groups were confirmed via microrheological investigations, assessing mucoadhesive properties of the thiolated derivative. The thiolation of the cyclodextrin did not affect the good cytocompatibility of the product. Interestingly, the cyclodextrin derivative forms small nanometric aggregates in an aqueous solution, both in the presence and absence of Dex, even at a low concentration (0.3%). The present study describes, therefore, a quick and easy microwave synthesis method, able to provide a highly thiolated and mucoadhesive 2-methyl-β-cyclodextrin with self-nano-aggregation properties, as a promising excipient to increase the bioavailability of the poorly soluble drug dexamethasone.

## Figures and Tables

**Table 1:** ^1^H NMR chemical shift (600 MHz, D_2_O, 25 °C, 5 mg/mL) of MβCD-SH, MβCD, and β-CD.

	MβCD-SH				MβCD		β-CD
	Unit C	Unit D	Unit A	Unit B	Unit A	Unit B	
H_1_	5.58	5.26	5.13	4.94	5.13	4.94	4.94
H_2_	3.18	3.47	3.26	3.53	3.26	3.53	3.52
H_3_	3.83	3.81	3.88	3.81	3.88	3.81	3.84
H_4_	3.55	3.60	3.48	3.46	3.48	3.45	3.46
H_5_	3.63	3.71	3.69	3.72	3.70	3.73	3.74
H_6/6′_	3.63	3.71	3.73	3.73	3.74	3.74	3.75
MeO	3.40	-	3.43	-	3.44	-	-

**Table 2:** Complex viscosity (η*), elastic modulus (G′), viscous modulus (G′′) of MβCD and MβCD-SH, with respect to the plain mucin dispersion. (* *p* < 0.05; ** *p* < 0.01 in respect of sample “Mucin”). Three independent experiments were performed and data are reported as average value ± standard deviation.

Sample	Complex Viscosity (cP)	G′ (Pa)	G′′ (Pa)
Mucin	0.573 ± 0.032	1.18 ± 0.20	6.32 ± 0.24
Mucin + MβCD	0.491 ± 0.043 *	1.08 ± 0.56	4.04 ± 0.53 **
Mucin + MβCD-SH	0.786 ± 0.08 *	1.24 ± 0.36	7.78 ± 0.27 **

**Table 3:** Size of the nanometric aggregates spontaneously formed in water solution. Values ± standard deviation.

Compound	Concentration (% *w*/*v*)	Aggregates Size ± Standard Deviation (nm)
MβCD	0.30 ÷ 12.50	not detectable aggregates
MβCD-SH	12.50	2.94 ± 0.08
MβCD-SH	5.00	2.86 ± 0.29
MβCD-SH	0.30	2.54 ± 0.42

## Supplementary Materials

The following supporting information can be downloaded at: https://www.mdpi.com/article/10.3390/polym14153170/s1, Figure S1. ^1^H NMR (600 MHz, DMSO-d_6_, 25 °C, 5 mg/mL) spectra of (a) MβCD and (b) MβCD-SH with integrated areas; Figure S2. HSQC (600 MHz, D_2_O, 25 °C) map of MβCD-SH (5 mg/mL); Figure S3. 1D ROESY (600 MHz, D_2_O, 25 °C, mix = 300 ms) spectra of anomeric protons of MβCD-SH (5 mg/mL); Figure S4. 1D TOCSY (600 MHz, D_2_O, 25 °C, mix = 80 ms) spectra of anomeric protons of MβCD-SH (5 mg/mL); Figure S5. COSY (600 MHz, D_2_O, 25 °C) map of MβCD-SH (5 mg/mL); Figure S6. ^1^H NMR (600 MHz, D_2_O, 25 °C) spectrum of MβCD-SH (5 mg/mL); Table S1 Microwave reaction conditions.: applied concentrations of cyclodextrin and thiourea, microwave setting (MW) of temperature and reaction time, results in terms of final yield and thiolation degree (TD) are reported. The selected conditions are reported in bold.

## References

[1] Liu H., Jiang W., Yang Z., Chen X., Yu D.G., Shao J. (2022). Hybrid Films Prepared from a Combination of Electrospinning and Casting for Offering a Dual-Phase Drug Release. Polymers.

[2] Kang S., Hou S., Chen X., Yu D.G., Wang L., Li X., Williams G.R. (2020). Energy-Saving Electrospinning with a Concentric Teflon-Core Rod Spinneret to Create Medicated Nanofibers. Polymers.

[3] Lv H., Guo S., Zhang G., He W., Wu Y., Yu D.G. (2021). Electrospun Structural Hybrids of Acyclovir-Polyacrylonitrile at Acyclovir for Modifying Drug Release. Polymers.

[4] Grassiri B., Zambito Y., Bernkop-Schnurch A. (2021). Strategies to prolong the residence time of drug delivery systems on ocular surface. Adv. Colloid Interface Sci..

[5] Pearson J.P., Chater P.I., Wilcox M.D. (2016). The properties of the mucus barrier, a unique gel—How can nanoparticles cross it?. Ther. Deliv..

[6] Lee D., Lu Q., Sommerfeld S.D., Chan A., Menon N.G., Schmidt T.A., Elisseeff J.H., Singh A. (2017). Targeted delivery of hyaluronic acid to the ocular surface by a polymer-peptide conjugate system for dry eye disease. Acta Biomater..

[7] Migone C., Mattii L., Giannasi M., Moscato S., Cesari A., Zambito Y., Piras A.M. (2020). Nanoparticles Based on Quaternary Ammonium Chitosan-methyl-beta-cyclodextrin Conjugate for the Neuropeptide Dalargin Delivery to the Central Nervous System: An In Vitro Study. Pharmaceutics.

[8] Leichner C., Jelkmann M., Bernkop-Schnurch A. (2019). Thiolated polymers: Bioinspired polymers utilizing one of the most important bridging structures in nature. Adv. Drug Deliv. Rev..

[9] Cesari A., Fabiano A., Piras A.M., Zambito Y., Uccello-Barretta G., Balzano F. (2020). Binding and mucoadhesion of sulfurated derivatives of quaternary ammonium-chitosans and their nanoaggregates: An NMR investigation. J. Pharm. Biomed. Anal..

[10] Hussain Asim M., Ijaz M., Rösch A.C., Bernkop-Schnürch A. (2020). Thiolated cyclodextrins: New perspectives for old excipients. Coord. Chem. Rev..

[11] Asim M.H., Nazir I., Jalil A., Matuszczak B., Bernkop-Schnürch A. (2020). Tetradeca-thiolated cyclodextrins: Highly mucoadhesive and in-situ gelling oligomers with prolonged mucosal adhesion. Int. J. Pharm..

[12] Asim M.H., Ijaz M., Mahmood A., Knoll P., Jalil A., Arshad S., Bernkop-Schnurch A. (2021). Thiolated cyclodextrins: Mucoadhesive and permeation enhancing excipients for ocular drug delivery. Int. J. Pharm..

[13] Asim M.H., Nazir I., Jalil A., Laffleur F., Matuszczak B., Bernkop-Schnurch A. (2020). Per-6-Thiolated Cyclodextrins: A Novel Type of Permeation Enhancing Excipients for BCS Class IV Drugs. ACS Appl. Mater. Interfaces.

[14] Grassiri B., Knoll P., Fabiano A., Piras A.M., Zambito Y., Bernkop-Schnurch A. (2022). Thiolated Hydroxypropyl-beta-cyclodextrin: A Potential Multifunctional Excipient for Ocular Drug Delivery. Int. J. Mol. Sci..

[15] Popovics-Tóth N., Tajti Á., Hümpfner E., Bálint E. (2020). Synthesis of 3,4-Dihydropyrimidin-2(1H)-one-phosphonates by the Microwave-Assisted Biginelli Reaction. Catalysts.

[16] Benesi H.A., Hildebrand J.H. (1949). A Spectrophotometric Investigation of the Interaction of Iodine with Aromatic Hydrocarbons. J. Am. Chem. Soc..

[17] Fabiano A., Piras A.M., Guazzelli L., Storti B., Bizzarri R., Zambito Y. (2019). Impact of Different Mucoadhesive Polymeric Nanoparticles Loaded in Thermosensitive Hydrogels on Transcorneal Administration of 5-Fluorouracil. Pharmaceutics.

[18] Dodero A., Williams R., Gagliardi S., Vicini S., Alloisio M., Castellano M. (2019). A micro-rheological and rheological study of biopolymers solutions: Hyaluronic acid. Carbohydr. Polym..

[19] Cesari A., Piras A.M., Zambito Y., Uccello Barretta G., Balzano F. (2020). 2-Methyl-beta-cyclodextrin grafted ammonium chitosan: Synergistic effects of cyclodextrin host and polymer backbone in the interaction with amphiphilic prednisolone phosphate salt as revealed by NMR spectroscopy. Int. J. Pharm..

[20] Cesari A., Recchimurzo A., Fabiano A., Balzano F., Rossi N., Migone C., Uccello-Barretta G., Zambito Y., Piras A.M. (2020). Improvement of Peptide Affinity and Stability by Complexing to Cyclodextrin-Grafted Ammonium Chitosan. Polymers.

[21] Piras A.M., Fabiano A., Chiellini F., Zambito Y. (2018). Methyl-beta-cyclodextrin quaternary ammonium chitosan conjugate: Nanoparticles vs macromolecular soluble complex. Int. J. Nanomed..

[22] Piras A.M., Zambito Y., Burgalassi S., Monti D., Tampucci S., Terreni E., Fabiano A., Balzano F., Uccello-Barretta G., Chetoni P. (2018). A water-soluble, mucoadhesive quaternary ammonium chitosan-methyl-beta-cyclodextrin conjugate forming inclusion complexes with dexamethasone. J. Mater. Sci. Mater. Med..

[23] Asim M.H., Moghadam A., Ijaz M., Mahmood A., Gotz R.X., Matuszczak B., Bernkop-Schnurch A. (2018). S-protected thiolated cyclodextrins as mucoadhesive oligomers for drug delivery. J. Colloid Interface Sci..

[24] Rao G.C.S., Ramadevi K., Sirisha K. (2014). Effect of β-cyclodextrin on Rheological Properties of some Viscosity Modifiers. Indian J. Pharm. Sci..

[25] Bansil R., Turner B.S. (2018). The biology of mucus: Composition, synthesis and organization. Adv. Drug Deliv. Rev..

[26] Messner M., Kurkov S.V., Brewster M.E., Jansook P., Loftsson T. (2011). Self-assembly of cyclodextrin complexes: Aggregation of hydrocortisone/cyclodextrin complexes. Int. J. Pharm..

[27] Messner M., Kurkov S.V., Jansook P., Loftsson T. (2010). Self-assembled cyclodextrin aggregates and nanoparticles. Int. J. Pharm..

[28] Muankaew C., Saokham P., Jansook P., Loftsson T. (2020). Self-assembly of cyclodextrin complexes: Detection, obstacles and benefits. Pharmazie.

[29] Jansook P., Kurkov S.V., Loftsson T. (2010). Cyclodextrins as solubilizers: Formation of complex aggregates. J. Pharm. Sci..

[30] Valente A.J., Carvalho R.A., Soderman O. (2015). Do Cyclodextrins Aggregate in Water? Insights from NMR Experiments. Langmuir.

[31] Loftsson T., Saokham P., Sa Couto A.R. (2019). Self-association of cyclodextrins and cyclodextrin complexes in aqueous solutions. Int. J. Pharm..

[32] Mazmanian K., Sargsyan K., Grauffel C., Dudev T., Lim C. (2016). Preferred Hydrogen-Bonding Partners of Cysteine: Implications for Regulating Cys Functions. J. Phys. Chem. B.

[33] Biswal H.S., Shirhatti P.R., Wategaonkar S. (2009). O−H···O versus O−H···S Hydrogen Bonding I: Experimental and Computational Studies on the p-Cresol·H2O and p-Cresol·H2S Complexes. J. Phys. Chem. A.

