# Supplementary material for: Thiolated 2-Methyl-β-Cyclodextrin as a Mucoadhesive Excipient for Poorly Soluble Drugs: Synthesis and Characterization
Source: Polymers (Basel). 2022 Aug 3;14(15):3170. doi: 10.3390/polym14153170 (PMC9370929; doi:10.3390/polym14153170)
Supplement: Supplementary file 1 [file polymers-14-03170-s001.zip › polymers-1847949-supplementary.pdf]

Supplementary information file of

Thiolated 2-methyl- β-cyclodextrin as a mucoadhesive excipient for poorly soluble drugs: synthesis and characterization

Brunella Grassiri[1], Andrea Cesari[2], Federica Balzano[2], Chiara Migone[1], Gergely.Kali[3], Andreas Bernkop-Schnürch[3], Gloria Uccello-Barretta[2], Ylenia Zambito[1,4], Anna Maria Piras[1]*

[1] 1 Department of Pharmacy, University of Pisa, Via Bonanno 33, 56126 Pisa, Italy;
[2] Department of Chemistry and Industrial Chemistry, University of Pisa, via Moruzzi 13, 56124 Pisa, Italy
[3] Center for Chemistry and Biomedicine, Department of Pharmaceutical Technology, Institute of Pharmacy,
[4] Interdepartmental Research Centre "Nutraceuticals and Food for Health", University of Pisa, Pisa, 56100, Italy
[5]University of Innsbruck, Innrain 80/82, A-6020 Innsbruck, Austria;
Corresponding author: anna.piras@unipi.it Tel.: +39 3392221213
Correspondence: e-mail@e-mail.com; Tel.: (optional; include country code; if there are multiple corresponding authors, add author initials)

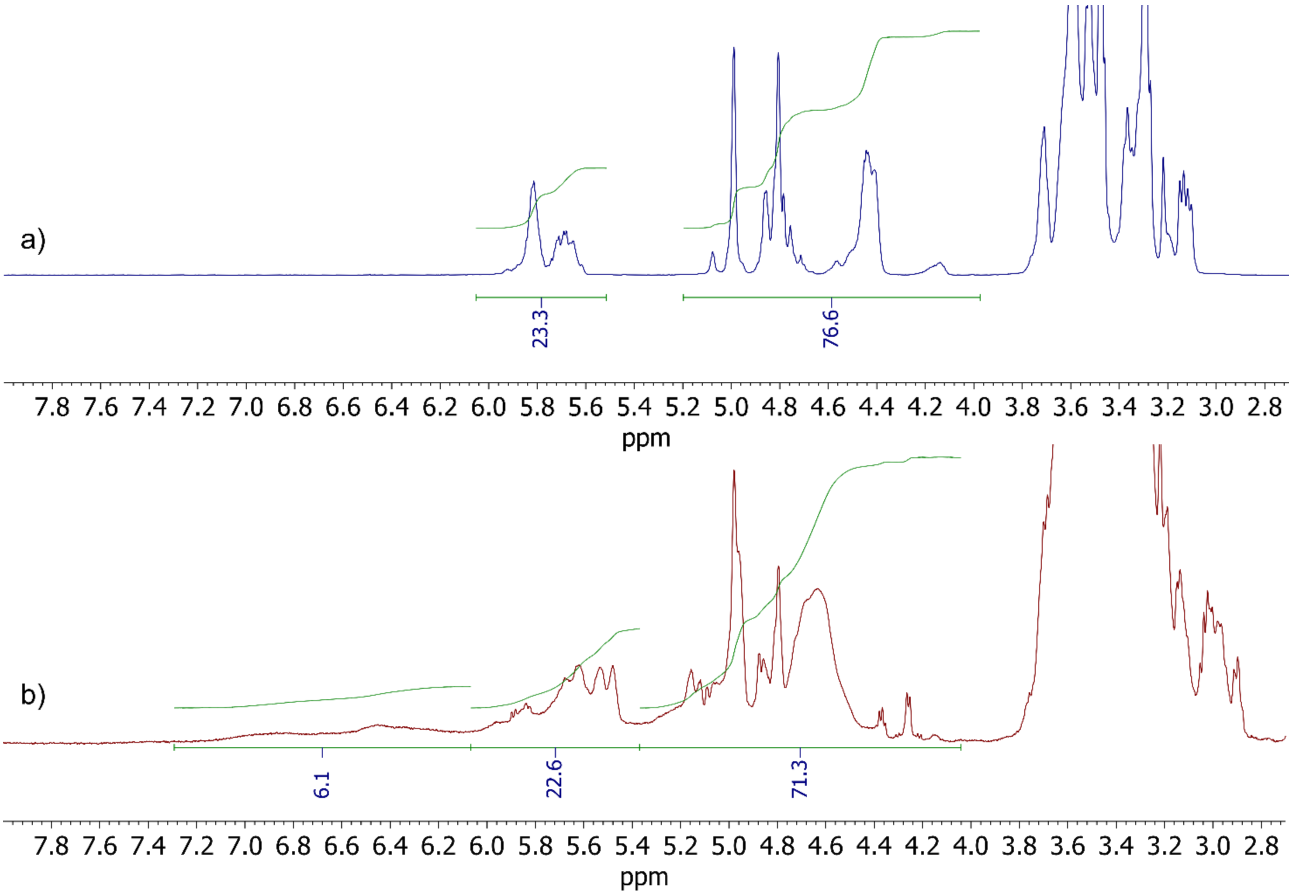

Figure S1. ^{1}H NMR (600 MHz, DMSO-d$_6$, 25 °C, 5 mg/mL) spectra of a) MβCD and b) MβCD-SH with integrated areas.

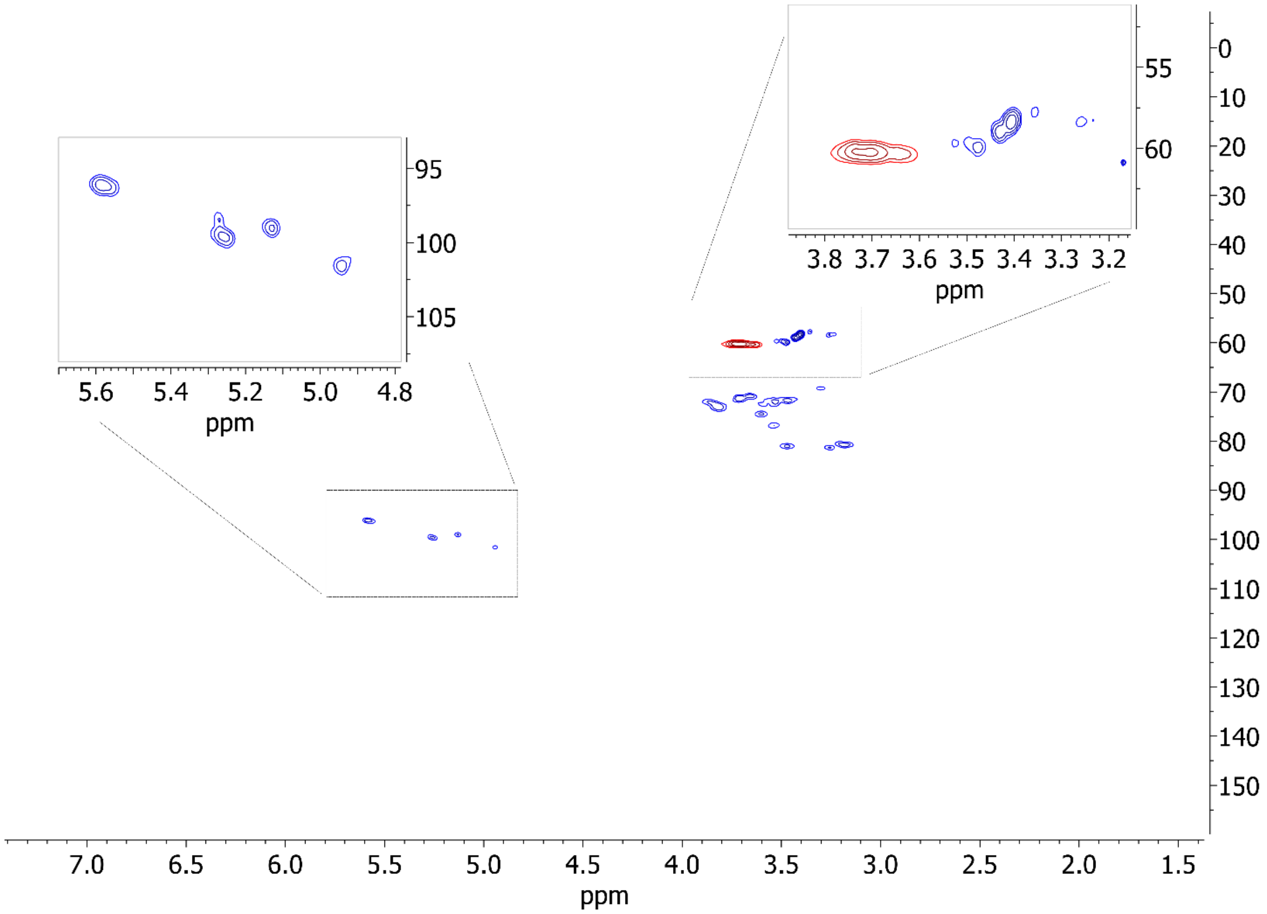

Figure S2. HSQC (600 MHz, D_2O, 25 °C) map of MβCD-SH (5 mg/mL).

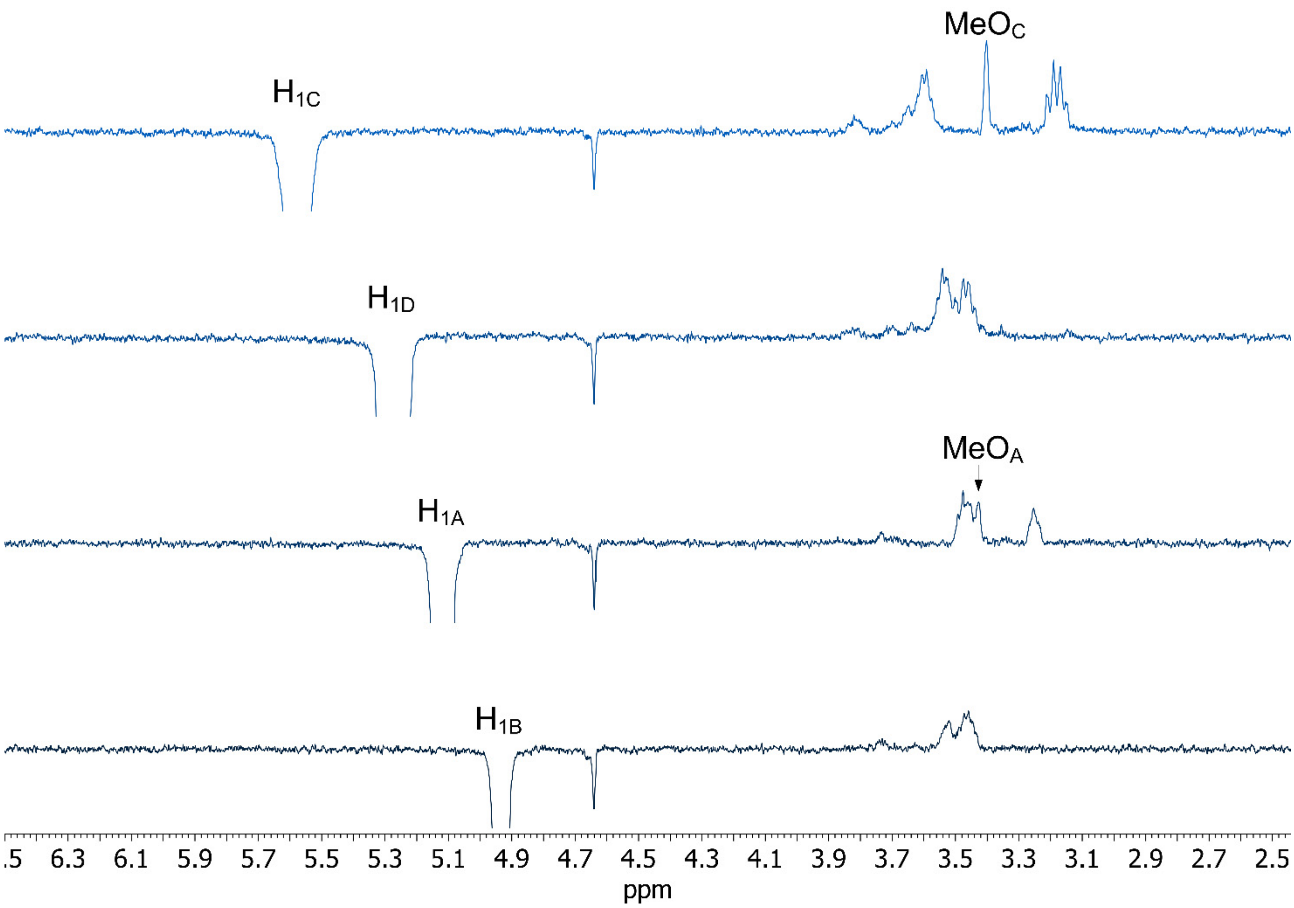

Figure S3. 1D ROESY (600 MHz, D_2O, 25 °C, mix=300 ms) spectra of anomeric protons of MβCD-SH (5 mg/mL).

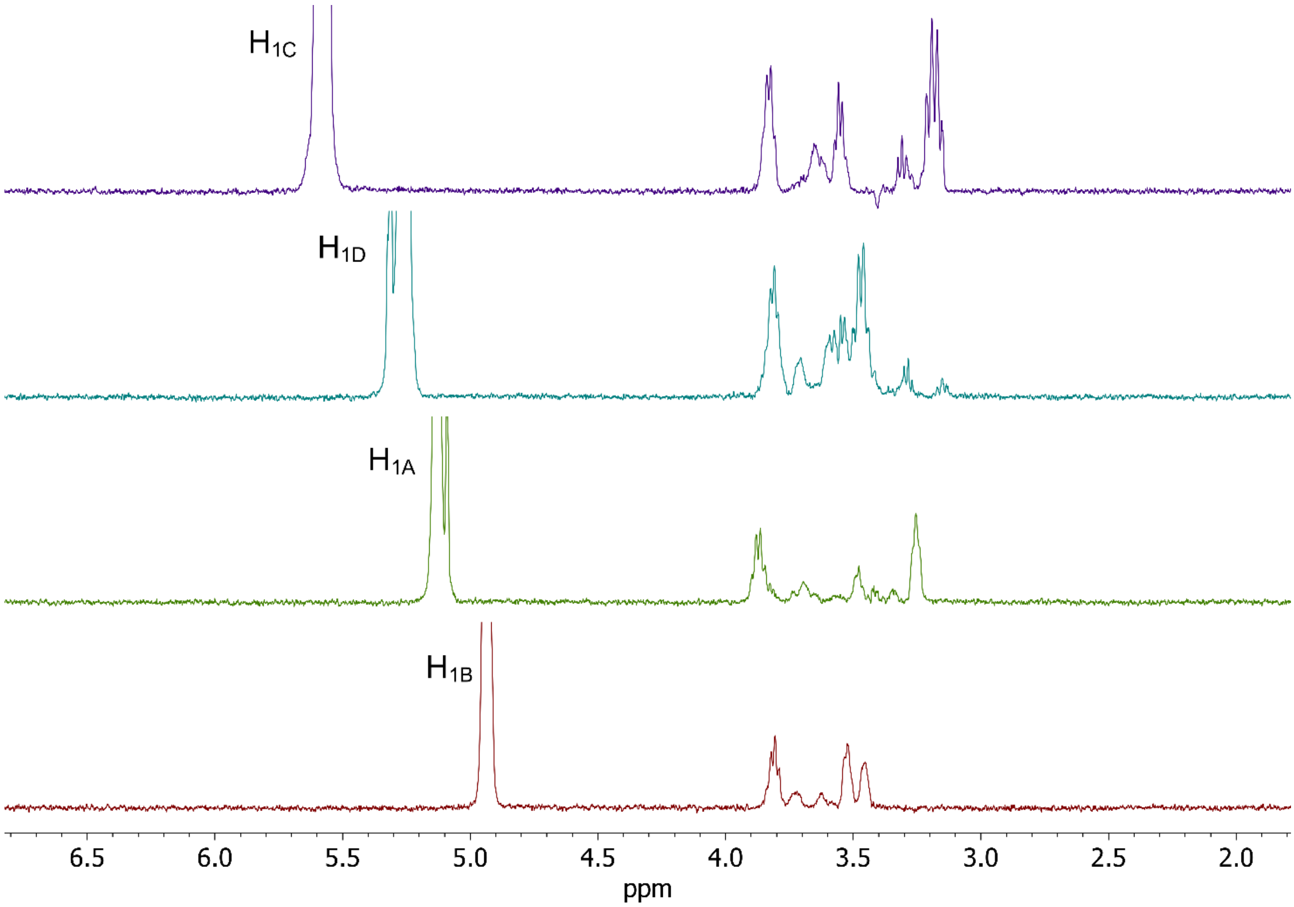

Figure S4. 1D TOCSY (600 MHz, D_2O, 25 °C, mix=80 ms) spectra of anomeric protons of MβCD-SH (5 mg/mL).

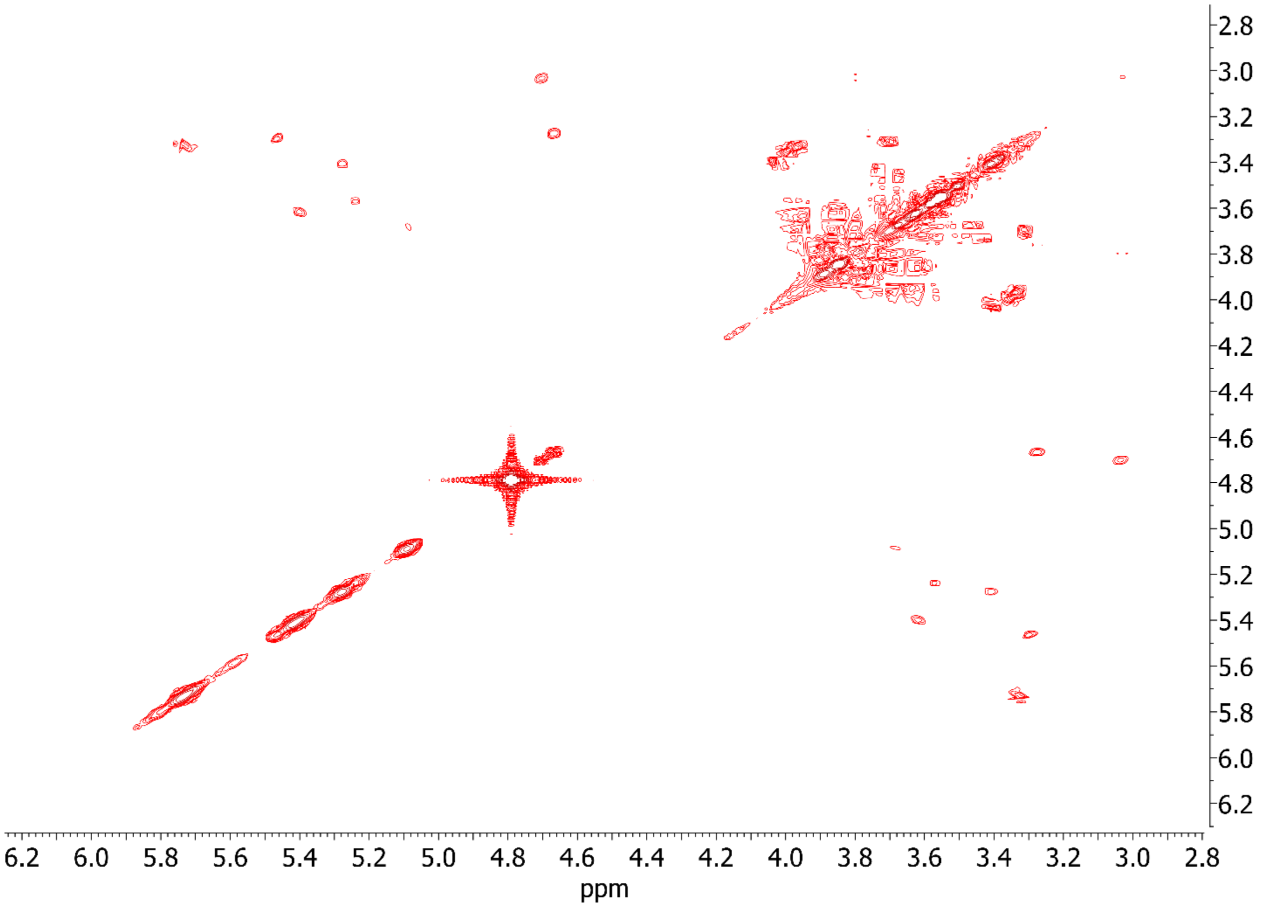

Figure S5. COSY (600 MHz, D_2O, 25 °C) map of MβCD-SH (5 mg/mL).

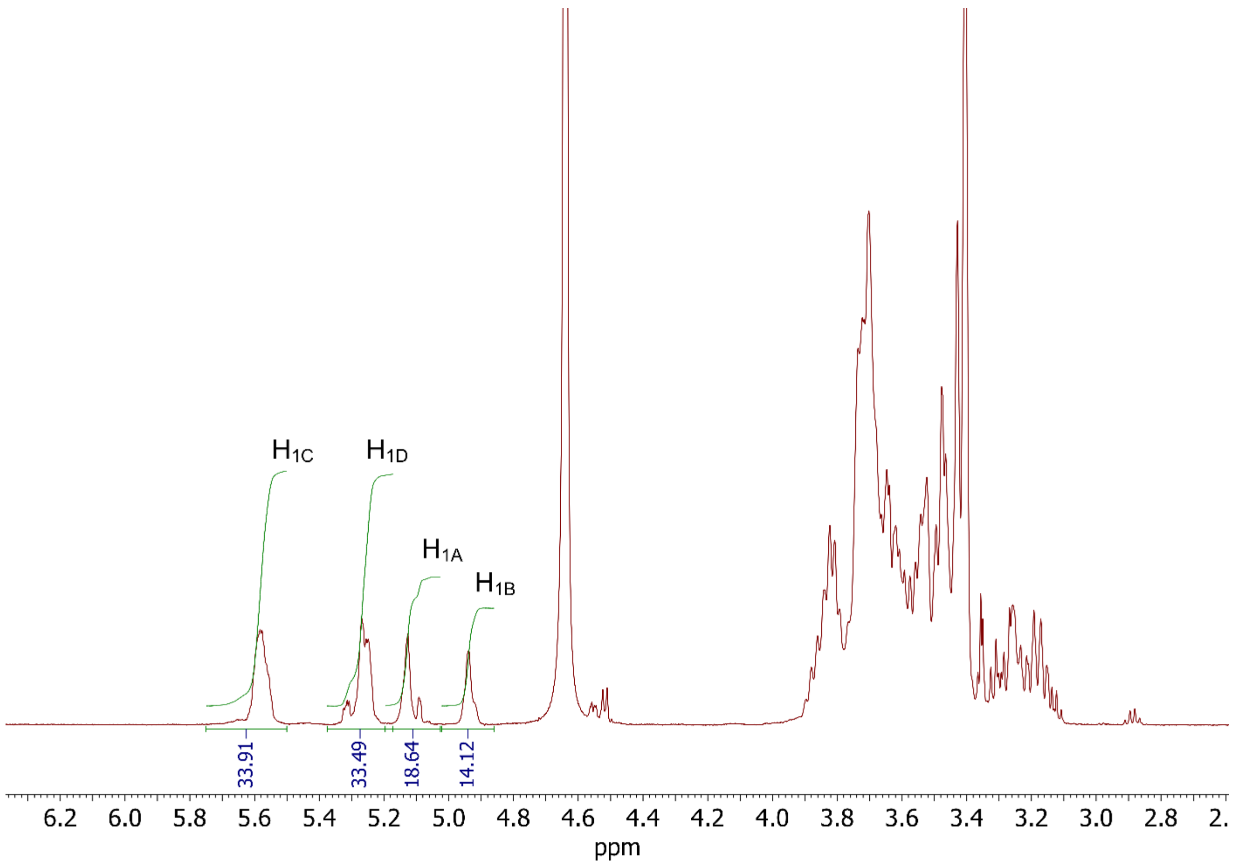

Figure S6. ^{1}H NMR (600 MHz, D_2O, 25 °C) spectrum of MβCD-SH (5 mg/mL).

Table S1 Microwave reaction conditions: applied concentrations of cyclodextrin and thiourea, microwave setting (MW) of temperature and reaction time, results in terms of final yield and thiolation degree (TD) are reported. The selected conditions are reported in bold.

**REACTION SETTING**					**RESULTS**	
**Cyclodextrin solution**		**Thiourea solution**		**MW conditions**	**TD**	**Yield**
**MβCD (mg/mL)**	**AcOH (M)**	**Thiourea (mg/mL)**	**HCl (M)**	**Temp. and reaction Time I and II step**	**(% sugars)**	**(%)**
40	0.174	214	0.44	80 °C (60 min)	15	37
40	1.74	214	0.44	87 °C (5min) - 80 °C (55min)	29	28
**40**	**1.74**	**214**	**0.44**	**87 °C (5 min) - 80 °C (110 min)**	**67**	**30**
40	1.74	428	0.44	87 °C (5min)-80 °C (55min)	51	2
40	0.174	214	0.10*	87 °C (5min)-80 °C (55min)	2	80

* Citrate Buffer
